# Takotsubo Cardiomyopathy Associated With the Usage of Nebulised Adrenaline for Acute Stridor: A Case Report

**DOI:** 10.7759/cureus.91550

**Published:** 2025-09-03

**Authors:** Haris Duvnjak, Matthew Wedlich, Daaniya Fatima, Babatunde Oremule, Alex Bowen

**Affiliations:** 1 Otolaryngology, Wrexham Maelor Hospital, Wrexham, GBR; 2 ENT, Northern Care Alliance, Manchester, GBR

**Keywords:** adrenaline, bilateral vocal cord paralysis, head and neck neoplasms, stridor, takotsubo cardiomyopathy (ttc)

## Abstract

A gentleman in his 70s with previously treated oropharyngeal squamous cell carcinoma presented acutely with worsening stridor. Flexible laryngoscopy on initial assessment demonstrated bilateral vocal cord palsy, and he was subsequently resuscitated with high-flow oxygen and nebulised adrenaline. On his third day of admission, antero-lateral T-wave inversions were incidentally found on a pre-operative electrocardiogram, and a transthoracic echocardiogram showed mid-apical akinesia. After a negative inpatient coronary angiogram, he was diagnosed with Takotsubo cardiomyopathy attributed to nebulised adrenaline therapy. This case report is the first to our knowledge to report an instance of this in a patient with acute airway obstruction caused by bilateral vocal cord palsy and no prior cardiac history.

## Introduction

Nebulised adrenaline, along with high-flow oxygen and other medical therapies such as intravenous dexamethasone, is commonly used as a part of resuscitating patients with acute stridor [1,2]. It is thought to cause vasoconstriction via α-adrenergic receptor agonism directly within the airway, thereby reducing mucosal oedema and helping relieve obstruction [3]. Although prolonged use of intravenous adrenaline is known to be associated with myocardial ischaemia via increased sympathetic activity and coronary vasospasm [4], nebulised administration causing Takotsubo cardiomyopathy (TTC) has been reported in the literature only once [5]. This case report is the first to our knowledge to report an instance of this in a patient with acute airway obstruction caused by bilateral vocal cord palsy with no previous cardiac history. Otolaryngologists and intensivists should be aware of the potential adverse effects associated with prolonged use of nebulised adrenaline.

## Case presentation

A gentleman in his 70s was admitted with 10 days of worsening stridor. He had a background of oropharyngeal squamous cell carcinoma (SCC) treated 12 years prior with chemoradiotherapy and a subsequent radiologically inserted gastrostomy for feeding.

Initial observations were within normal range, including pulse oximetry of 98% on room air. On examination, the patient had mild inspiratory stridor with no use of accessory muscles. Flexible nasoendoscopy showed bilaterally fixed vocal cords in adduction with a narrowed posterior airway in keeping with bilateral vocal cord palsy. Mild cord erythema was seen with no oedema or exudates.

**Figure 1:**
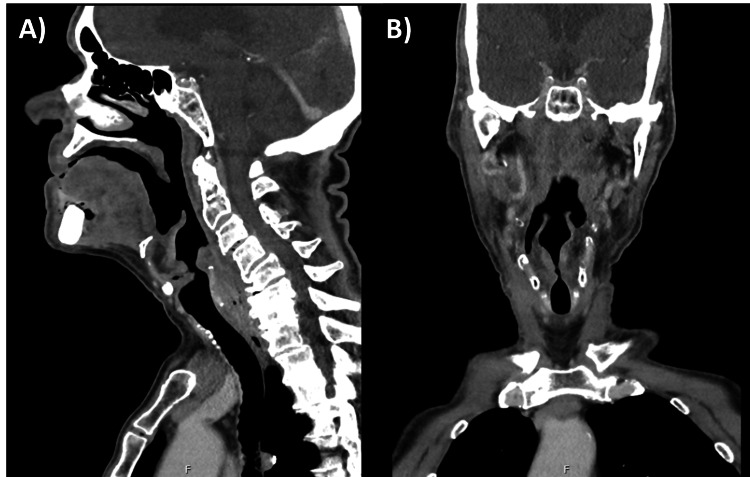
CT neck with contrast in sagittal (A) and coronal (B) planes demonstrating an upper oesophageal sphincter mass lesion with oedematous changes, suspicious for underlying malignancy.

C-reactive protein on admission was 10.6 (range 0-9.9) and white cell count 8.8 (range 5.5-11.0). Virology was negative for SARS-CoV-2, respiratory syncytial virus and influenza. Computed tomography (CT) with contrast of his neck showed an upper oesophageal sphincter mass lesion, initially reported as oedema, and necrotic left-sided cervical lymph node metastasis, suspicious for underlying malignancy (Figure 1).

After review by anaesthetics and intensive care teams, a joint decision was made to admit the patient under otolaryngology to the high-dependency unit for airway observation. The patient received nebulised adrenaline 1 mL (one in 1,000) every 12 hours and intravenous dexamethasone 6.6 mg every eight hours.

The surgical plan was for panendoscopy + biopsy +/- tracheostomy to be done following the weekend, as the patient's airway was stable.

Investigations

**Figure 2:**
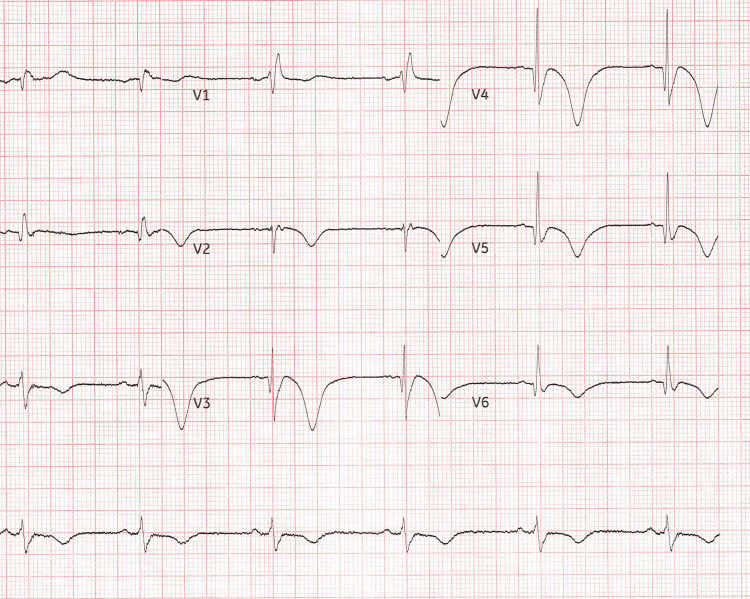
Scanned pre-operative ECG trace showing T-wave inversions and right bundle branch block as described. Paper output speed: 25 mm/s. Voltage calibration: 10.0 mm/mV.

Electrocardiogram (ECG) on day 3, part of his pre-operative anaesthetic review, showed progressive T-wave inversion in leads II, III, aVF, V5 and V6 compared to his admission ECG, as well as right bundle branch block, which was consistent with an ECG two months prior (Figure 2). Troponin I was performed following this, which was 492 (range 0-54), which was concerning for acute coronary syndrome (ACS).

Differential diagnoses

The main cause of ACS in this case was non-ST-segment elevation myocardial infarction (NSTEMI), which was excluded by angiography. A non-cardiac differential diagnosis was pulmonary embolism; this, however, was not suspected due to the absence of chest pain, tachypnoea and typical ECG findings (sinus tachycardia, S1Q3T3, P pulmonale and right axis deviation) [6].

The differential diagnosis for bilateral vocal cord palsy is broad. However, with a history of prior head and neck SCC and necrotic cervical lymph nodes, underlying malignancy was the main pathology to be excluded. As the patient had radiotherapy to this anatomical area previously, post-radiotherapy scarring with subsequent arytenoid joint fixation was a less urgent but plausible differential diagnosis.

Management

Although the patient denied chest pain, he was initially started on dual-antiplatelet therapy (DAPT) following discussion with cardiology, given the high troponin and ECG findings in keeping with NSTEMI. He was booked for a transthoracic echocardiogram (TTE) and an inpatient coronary angiogram. His adrenaline nebuliser was omitted. A repeat troponin done six hours after trended downwards to 316.

**Figure 3:**
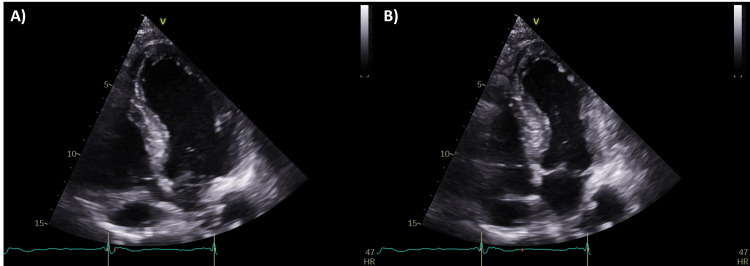
Snapshots from apical four-chamber transthoracic echocardiogram (TTE) at end diastole (A) and end systole (B), demonstrating good basal contraction with impaired mid-apical function.

Panendoscopy performed later during the week demonstrated a hypopharyngeal mass not highlighted on the initial CT report, with a biopsy performed. Tracheostomy was also performed to secure the patient’s airway. TTE on day 4 demonstrated an inspired left ventricular ejection fraction (LVEF) of 40-45% with mid-apical akinesia (Figure 3). A coronary angiogram was done on day 5 and demonstrated small-calibre but unobstructed epicardial coronary arteries, with a dominant right coronary artery.

Upon further cardiology assessment, the patient’s DAPT were stopped, and a repeat TTE one week later was unchanged. Given the angiographic findings of normal coronary arteries together with the TTE findings, the final diagnosis was TTC, attributed to the use of an adrenaline nebuliser and acute bodily stress. No further cardiac treatment was initiated as troponin levels were improving and cardiac function was stable.

Complications and outcome

The patient continued to be managed on a surgical ward prior to discharge. He received training on routine tracheostomy care, including maintaining inner tube hygiene, suction and humidification.

The patient developed acute rectal bleeding on day 15, likely provoked by DAPT therapy. This was managed conservatively, and an inpatient colonoscopy demonstrated colonic telangiectasia and diverticulosis with no active bleeding.

Subsequent histology showed hypopharyngeal SCC. His imaging was reviewed at the following multidisciplinary team discussion, and his tumour was staged at T4bN1M0 (TNM system). Due to his previous SCC and locally invasive malignancy, he was not suitable for further radiotherapy and was referred for palliative immunotherapy.

## Discussion

TTC, also known as stress-induced cardiomyopathy, is an acute and reversible heart failure syndrome often triggered by intense emotional or physical stress, leading to temporary dysfunction of the left ventricle. This condition can mimic ACS, but without obstructive coronary artery disease. It is the underlying aetiology of 1-2% of all cases that present with ACS. Although it is a transient condition with excellent prognosis in its later period, mortality rates are significant early during the disease course (1-1.5% of cases). Its pathophysiology is not fully understood, but adrenaline has been identified to play a key role as a trigger, as seen in endogenous adrenergic surges and catecholamine storms associated with neuroendocrine tumours. One theory states that due to a higher concentration of β-adrenergic receptors in the left ventricular apex relative to the base, there is an increased response to high levels of circulating catecholamines, therefore resulting in its characteristic apical akinesia [7].

Clinical features of TTC include sudden-onset chest pain, dyspnoea, and electrocardiographic changes resembling ACS, such as ST-segment elevation, T-wave depression and left bundle branch block. In terms of cardiac enzymes, rises in troponin are seen, though typically less compared to ST-segment elevation myocardial infarction. The diagnosis is confirmed with invasive coronary angiography, which should demonstrate non-obstructive coronary arteries. TTE is useful in supporting the diagnosis by demonstrating the typical findings of apical to mid-ventricular akinesis with basal hyperkinesis [8].

Regarding the management of TTC, clinical guidelines emphasise the importance of diagnosing and differentiating it from other cardiac conditions, such as myocardial infarction, as treatment strategies differ. The use of beta-blockers is often recommended to manage symptoms and reduce adrenergic stimulation in TTC patients, especially in the acute phase. The management of stress-induced cardiomyopathy usually involves supportive care, with a focus on addressing the underlying stressor, symptom relief, and ensuring that any reversible triggers (such as medication) are minimised [9]. Best practice is predominantly based on clinical experience and international expert consensus (e.g., Ghadri et al. [10]).

Adrenaline is commonly used therapeutically in emergency settings for conditions such as stridor or anaphylaxis. Its sympathomimetic effects can significantly increase heart rate, blood pressure, and myocardial oxygen demand. It is established from previous cases and pre-clinical studies that intravenous administration can potentially trigger TTC, particularly in susceptible individuals with pre-existing cardiovascular disease [8]. The literature regarding nebulised adrenaline, however, remains limited, with only one other clinical case acting as the primary source of information [5]. Compared to this case, our patient was similar in age (70 versus 66 years) but had different malignancy characteristics (hypopharyngeal T4bN1M0 versus laryngeal T3N2cM0) and duration in adrenaline therapy (seven versus two doses). 

In the context of upper airway obstruction, the benefits of nebulised adrenaline in reducing mucosal oedema and improving oxygenation usually outweigh potential cardiac adverse effects. However, the limited data surrounding the use of nebulised adrenaline in these patients suggests that it should be used cautiously, particularly in individuals with known cardiac vulnerabilities. The authors therefore suggest that clinicians, such as emergency physicians, intensivists, internists and otolaryngologists, should be aware of these risks and reassess patients receiving this therapy regularly to rationalise its use. The authors also suggest performing a baseline ECG prior to initiating nebulised adrenaline, as well as initially investigating suspected ACS with serial ECG and cardiac enzymes. Further research in the form of large-scale prospective and experimental studies is needed to comprehensively understand the relationship between nebulised adrenaline and stress cardiomyopathy.

## Conclusions

This rare case demonstrates that while nebulised adrenaline is effective in managing airway conditions like stridor, its prolonged use can have significant adverse cardiac effects. Emergency physicians, internists, intensivists and otolaryngologists should be aware of these, including TTC, as well as tachycardia, hypertension and arrhythmia. We advise clinicians to carefully consider any previous cardiac history and perform a baseline ECG prior to administration, as well as serial ECG and cardiac enzymes for patients in whom myocardial ischaemia or TTC is suspected.
